# The distribution and pathogenic risk of non‐9‐valent vaccine covered HPV subtypes in cervical lesions

**DOI:** 10.1002/cam4.4532

**Published:** 2022-01-03

**Authors:** Mingjun Ma, Jingfen Zhu, Yongbin Yang, Xiaoyun Wang, Yubiao Jin, Jiawen Zhang, Sufang Wu

**Affiliations:** ^1^ Department of Obstetrics and Gynecology Shanghai General Hospital School of Medicine Shanghai Jiao Tong University Shanghai P.R. China; ^2^ School of Public Health Shanghai Jiao Tong University Shanghai P.R. China; ^3^ Department of Pathology Shanghai General Hospital School of Medicine Shanghai Jiao Tong University Shanghai P.R. China; ^4^ Reproductive Medicine Center Department of Obstetrics and Gynecology Shanghai General Hospital School of Medicine Shanghai Jiao Tong University Shanghai P.R. China

**Keywords:** cervical lesions, CIN, HPV genotypes, HPV vaccine, risk factors

## Abstract

Human papillomavirus (HPV) infection is the main cause of female precancerous lesions and cervical cancer. The development and application of HPV prophylactic vaccines have been recognized as a major effective intervention for the control of cervical lesions. However, the infection rate and clinical characters of non‐9‐valent vaccine covered HPV subtypes are still worth studying. In this retrospective study, we included patients diagnosed and treated in the Department of Gynecology of Shanghai General Hospital between January 2017 and February 2021. The clinical features of non‐9‐valent vaccine covered HPV subtypes were explored in 2179 patients who have normal results, 338 patients with cervical intraepithelial neoplasia 1 (CIN1), and 153 patients with ≥CIN2. Univariate analysis showed that compared to the normal cervix group, age ≥50, pregnancy ≥5, delivery ≥3, menopause, no condom use, and cervical transformation zone type III were risk factors for CIN1 or ≥CIN2 (*p* < 0.05). Thirty‐one percent of CIN1 and 26% of ≥CIN2 were attributed to HPV51, HPV53, HPV56, and HPV68. Multivariate analysis revealed that HPV53, HPV81, age, menopause, cervical transformation area and involved glands were independent risk factors for ≥CIN2 group compared to the CIN1 group (*p* < 0.05). Additionally, among the 14 non‐9‐valent vaccine covered HPV subtypes, the infection rates of HPV53, 56, 51, and 68 were higher in this study. In conclusion, our study demonstrated the distribution and pathogenic risk of non‐9‐valent vaccine covered HPV subtypes in cervical lesions. These findings might supply a foundation for optimizing cervical cancer prevention in the post‐vaccine era.

## Graphical abstract

We identified the most non‐9‐valent vaccine covered carcinogenic HPV types, so as to provides a basis for further optimizing screening methods and eliminating cervical cancer in the post‐vaccine era.

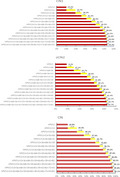

## 1 INTRODUCTION

Cervical cancer is the fourth most common malignancy among women, in the light of a report using the GLOBOCAN 2018 database, there were 569,847 new cases of cervical cancer among 311,365 deaths globally in 2018.^1^ Persistent human papillomavirus (HPV) infection, especially high‐risk HPV (HR‐HPV), is a major cause of precancerous lesions and cervical cancer.^2^ It has been estimated that more than 50–80% of sexually active women will be infected with one or more types of genital HPV in their lifetime.^3^ The emergence and application of HPV preventive vaccines have been recognized as a major advancement and the most effective intervention for the control of cervical lesions.^4^

Presently, 9‐valent vaccine covering HPV6/11/16/18/31/33/45/52/58 has been used in China. VIVIANE, FUTURE III, and other studies have confirmed the preventive effect of HPV vaccine on cervical cancer,^5^, ^6^, ^7^ and the number of deaths among vaccinated women might be reduced by >4 million over the next decade.^8^ However, the 9‐valent vaccines do not encompass all HR‐HPVs, which might cause cervical lesions, and the distribution of HPV genotypes varies between regions and countries, leading to geographical‐based changes in the incidence and mortality of cervical cancer.^9^, ^10^ Previous VIVIANE studies (bivalent vaccine) and FUTURE III studies (tetravalent vaccine) have not found the protective effect of the vaccine on cervical intraepithelial neoplasia (CIN) 2+ lesions.^5^, ^7^ A recent meta‐analysis showed that in the post‐vaccine era, the infection rate of some non‐vaccine coverage types also increased significantly.^11^ In addition, the female vaccination coverage must be at least 50% to have a protective effect on unvaccinated population.^12^ Therefore, the existing multivalent HPV vaccines cannot completely prevent cervical cancer. The majority of studies have focused on cervical cancer screening and vaccine effectiveness, whereas few studies analyzed the infection rate and prevalence of non‐9‐valent vaccine covered HPV subtypes, as well as the clinical characteristics and risk factors of non‐9‐valent vaccine covered HPV‐ infected patients.

In this retrospective study, we studied the non‐9‐valent vaccine covered HPV subtypes in cervical lesions, analyzed their distribution and the clinical characteristics of these HPV‐infected patients. We also identified the most non‐9‐valent vaccine covered carcinogenic HPV types and the risk factors of CIN patients.

## 2 MATERIAL AND METHODS

### 2.1 Patient samples

The clinical data of 7400 patients treated at the Department of Gynecology of Shanghai General Hospital from January 2017 to February 2021 were analyzed in this retrospective study. All cases provided cervical histopathological results and pre‐treatment HPV typing results. According to the inclusion or exclusion criteria, 2670 patients with non‐9‐valent vaccines covered HPV subtype infections were selected for analysis. The patients were divided into normal group (2179 cases), CIN1 group (338 cases), and ≥CIN2 (including CIN2 and CIN3 and carcinoma in situ) group (153 cases). The HPV typing data, histopathological data, and age were collected from each group.

The inclusion criteria were as follows: (1) cervical HPV and cytology sampling qualified; (2) colposcopy was performed in the gynecological clinic of our hospital; (3) HPV classification, ThinPrep cytologic test (TCT), Pathological results of colposcopy biopsy, and baseline data were obtained. Exclusion criteria were as follows: (1) previous hysterectomy or conization; (2) women infected with any type of the 9‐valent vaccine covered HPV; (3) other malignant tumors.

### 2.2 HPV detection and typing methods

The PCR‐reverse dot‐hybridization technology was used for HPV detection using a PCR‐RDB HPV genotyping assay (Yaneng Bioscience Co., Ltd., China),^13^ it can detect a total of 23 HPV types, including 17 HR‐HPV subtypes (16, 18, 31, 33, 35, 39, 45, 51, 52, 53, 56, 58, 59, 66, 68, 73, and 82), 6 LR‐HPV subtypes (6, 11, 42, 43, 81, and 83). Among these subtypes, 14 types were non‐9‐valent vaccine covered HPV subtypes (35, 39, 51, 53, 56, 59, 66, 68, 73, 82, 42, 43, 81, 83). Follow the kit instructions for experimental operation and data analysis.

### 2.3 TCT detection

Cytological classification of the samples was performed by two experienced pathologists based on the Bethesda System Standard 2001 in a double‐blinded manner.

### 2.4 Colposcopy and pathological examination

The diagnosis of cervical diseases in all patients was based on cervical histopathology. The cervical tissue is obtained from colposcopy biopsy or surgical submission specimens. The histopathological results of the highest level of the cervix were viewed as the final diagnosis of the disease. The specimens were processed using the standard histopathological methods and evaluated by at least two pathologists. All colposcopy operations were performed by qualified colposcopy specialists at our Center. The pathology results were classified as normal, CIN1, CIN2, CIN3, and carcinoma in situ.

### 2.5 Statistical analysis

The clinical characteristics and multiple HPV infections of the patients were tested by chi‐square test. For samples with multiple HPV infections, the infection of one HPV subtype contributed to the occurrence of the disease as a partial attribution. The prevalence rate of HPV genotypes and the attribution rate of cervical lesions are based on the literature,^14^ the “attributive factor” of a certain HPV subtype is calculated by the formula “the number of cases of a single HPV subtype infection in the disease/the number of cases of any HPV subtype single infection.” Thus, the attribution rate of a specific HPV subtype of the disease is as follows: (the number of single infections of HPV subtype + multiple infections of an HPV subtype × attribution factor)/the total number of cases of the disease. The correlation between HPV subtypes and cervical diseases was analyzed by binary logistic regression, and 95% is considered the confidence interval. A *p* < 0.05 indicate a statistical difference significantly. All statistical analyses were performed using software package of social science statistical software version 26 (SPSS, IBM Co., Armonk, NY, USA).

## 3 RESULTS

### 3.1 Analysis of clinical characteristics of patients infected with non‐9‐valent vaccine covered HPV subtypes

Among the three groups of patients infected with non‐9‐valent vaccine covered HPV subtypes, CIN1 group had the highest median age (48 years), and ≥CIN2 group had the highest number of pregnancies and parities, with an average of 2.99 and 1.52, respectively. The univariate analysis was used to compare the clinical variables. Compared to the normal group, age ≥50 years, pregnancy ≥5 times, parity ≥3 times, menopause, no condom use, cervical transformation zone type III, and HPV‐negative were risk factors for CIN1 or ≥CIN2 (*p* < 0.05) (Table 1).

**TABLE 1:** Clinical characteristics of patients with non‐9‐valent vaccine covered HPV infection

Variables	Normal (*n* = 2179)	CIN1 (*n* = 338)	*p*	Normal (*n* = 2179)	≥CIN2 (*n* = 153)	*p*
	*n* (%)	*n* (%)		*n* (%)	*n* (%)	
Age						
Median (years)	40	48		40	43	
<50	1691 (78)	181 (54)	<0.0001	1691 (78)	106 (69)	0.018
≥50	488 (22)	157 (46)		488 (22)	47 (31)	
Pregnancy						
Mean (times)	2.48	2.65		2.48	2.99	
<5	1990 (91)	298 (88)	0.06	1990 (91)	128 (84)	0.001
≥5	189 (9)	40 (12)		189 (9)	25 (16)	
Parity						
Mean (times)	1.20	1.34		1.20	1.52	
<3	2064 (95)	307 (91)	0.004	2064 (95)	138 (90)	0.018
≥3	115 (5)	31 (9)		115 (5)	15 (10)	
Condom						
Yes	631 (29)	70 (21)	0.002	631 (29)	30 (20)	0.013
No	1548 (71)	268 (79)		1548 (71)	123 (80)	
Menopause						
Yes	383 (18)	138 (41)	<0.0001	383 (18)	54 (35)	<0.001
No	1796 (82)	200 (59)		1796 (82)	99 (65)	
Cervical transformation area						
Type I	254 (12)	18 (5)	<0.0001	254 (12)	9 (6)	<0.001
Type II	781 (36)	72 (21)		781 (35)	33 (22)	
Type III	1144 (52)	248 (74)		1144 (53)	111 (72)	
HPV						
Positive	1705 (78)	238 (70)	0.001	1705 (78)	100 (65)	<0.001
Negative	474 (22)	100 (30)		474 (22)	53 (35)	

### 3.2 Analysis of infection in single‐ and multi‐non‐9‐valent vaccine covered HPV subtypes

Among 14 non‐9‐valent vaccine covered HPV subtypes, the infection rates of HPV53, 56, 51, and 68 were higher in this study: 17.6%, 13.2%, 12.6%, and 9.3%, respectively. At the same time, the infection rate of HPV81 was high (4.23%) in polymorphic infections. In CIN1 group, the infection rates of HPV53, 56 and 51 were higher: 14.3%, 14.2%, and 12.4%, respectively. However, among the patients with multi‐type HPV infection, the infection rates of HPV35 and 81 were second only to HPV53, accounting for 4.14%. In patients with ≥CIN2, the infection rates of HPV56 (13.1%), 51 (11.8%) and 53 (10.5) were higher, and the subtypes with high monotype HPV infection rates were 51 (8.5%), 68 (5.2%), and 53 and 56 (4.6%). However, in polymorphic infections, the infection rate of HPV35 and 39 was higher than that of HPV53 (Table 2).

**TABLE 2:** Analysis of infection in single‐ and multi‐type non‐9‐valent vaccine covered HPV subtypes

HPV types	Normal (*n* = 2179)			CIN1 (*n* = 338)			≥CIN2 (*n* = 153)			Total		
	Positive *n* (%^a^)	Single type *n* (%^a^)	Multiple infections *n* (%^a^)	Positive *n* (%^a^)	Single type *n* (%^a^)	Multiple infections *n* (%^a^)	Positive *n* (%^a^)	Single type *n* (%^a^)	Multiple infections *n* (%^a^)	Positive *n* (%^a^)	Single type *n* (%^a^)	Multiple infections *n* (%^a^)
HPV35	94 (4.3)	52 (2.4)	42 (1.9)	30 (8.9)	16 (4.7)	14 (4.1)	15 (9.8)	4 (2.6)	11 (7.2)	139 (5.2)	72 (2.7)	67 (2.5)
HPV39	190 (8.7)	116 (5.3)	74 (3.4)	21 (6.2)	9 (2.7)	12 (3.6)	13 (8.5)	2 (1.3)	11 (7.2)	224 (8.4)	127 (4.8)	97 (3.6)
HPV42	120 (5.5)	43 (2.0)	77 (3.5)	13 (3.9)	3 (0.9)	10 (3.0)	10 (6.5)	3 (2.0)	7 (4.6)	143 (5.4)	49 (1.8)	94 (3.5)
HPV43	109 (5.0)	42 (1.9)	67 (3.1)	10 (3.0)	0 (0.0)	10 (3.0)	5 (3.3)	1 (0.7)	4 (2.6)	124 (4.6)	43 (1.6)	81 (3.0)
HPV51	275 (12.6)	167 (7.7)	108 (5.0)	42 (12.4)	30 (8.9)	12 (3.6)	18 (11.8)	13 (8.5)	5 (3.3)	335 (12.6)	210 (7.9)	125 (4.7)
HPV53	405 (18.6)	274 (12.6)	131 (6.0)	50 (14.8)	31 (9.2)	19 (5.6)	16 (10.5)	7 (4.6)	9 (5.9)	471 (17.6)	312 (11.7)	159 (6.0)
HPV56	283 (13.0)	180 (8.3)	103 (4.7)	48 (14.2)	18 (5.3)	30 (8.9)	20 (13.1)	7 (4.6)	13 (8.5)	351 (13.2)	205 (7.7)	146 (5.5)
HPV59	160 (7.3)	80 (3.7)	80 (3.7)	24 (7.1)	14 (4.1)	10 (3.0)	7 (4.6)	4 (2.6)	3 (2.0)	191 (7.2)	98 (3.7)	93 (3.5)
HPV66	173 (7.9)	105 (4.8)	68 (3.1)	27 (8.0)	14 (4.1)	13 (3.9)	7 (4.6)	2 (1.3)	5 (3.3)	207 (7.8)	121 (4.5)	86 (3.2)
HPV68	202 (9.3)	123 (5.6)	79 (3.6)	30 (8.9)	17 (5.0)	13 (3.9)	15 (9.8)	8 (5.2)	7 (4.6)	247 (9.3)	148 (5.5)	99 (3.7)
HPV73	44 (2.0)	18 (0.8)	26 (1.2)	7 (2.1)	3 (0.9)	4 (1.2)	3 (2.0)	1 (0.7)	2 (1.3)	54 (2.0)	22 (0.8)	32 (1.2)
HPV81	162 (7.4)	72 (3.3)	90 (4.1)	21 (6.2)	7 (2.1)	14 (4.1)	12 (7.8)	3 (2.0)	9 (5.9)	195 (7.3)	82 (3.1)	113 (4.2)
HPV82	26 (1.2)	12 (0.6)	14 (0.6)	8 (2.4)	2 (0.6)	6 (1.8)	6 (3.9)	3 (2.0)	3 (2.0)	40 (1.5)	17 (0.6)	23 (0.9)
HPV83	11 (0.5)	2 (0.1)	9 (0.4)	3 (0.9)	1 (0.3)	2 (0.6)	2 (1.3)	0 (0.0)	2 (1.3)	16 (0.6)	3 (0.1)	13 (0.5)

^a^ HPV subtype infection rate was calculated by dividing the number of women infected with HPV by the total number of participants in the study.

Additionally, among all patients, the infection rates of HPV53, HPV51, HPV56, and HPV39 were 10.1%, 7.5%, 6.1%, and 4.4%, respectively. The higher infection rates of single HPV were 53, 51, 56, and 68. In the multi‐type HPV infection, the infection rate of HPV42 was next to that of HPV51, that is, 3.04% (Table S1).

### 3.3 Cumulative attribution rate of 14 non‐9‐valent vaccine covered HPV subtypes in cervical lesions

**FIGURE 1:**
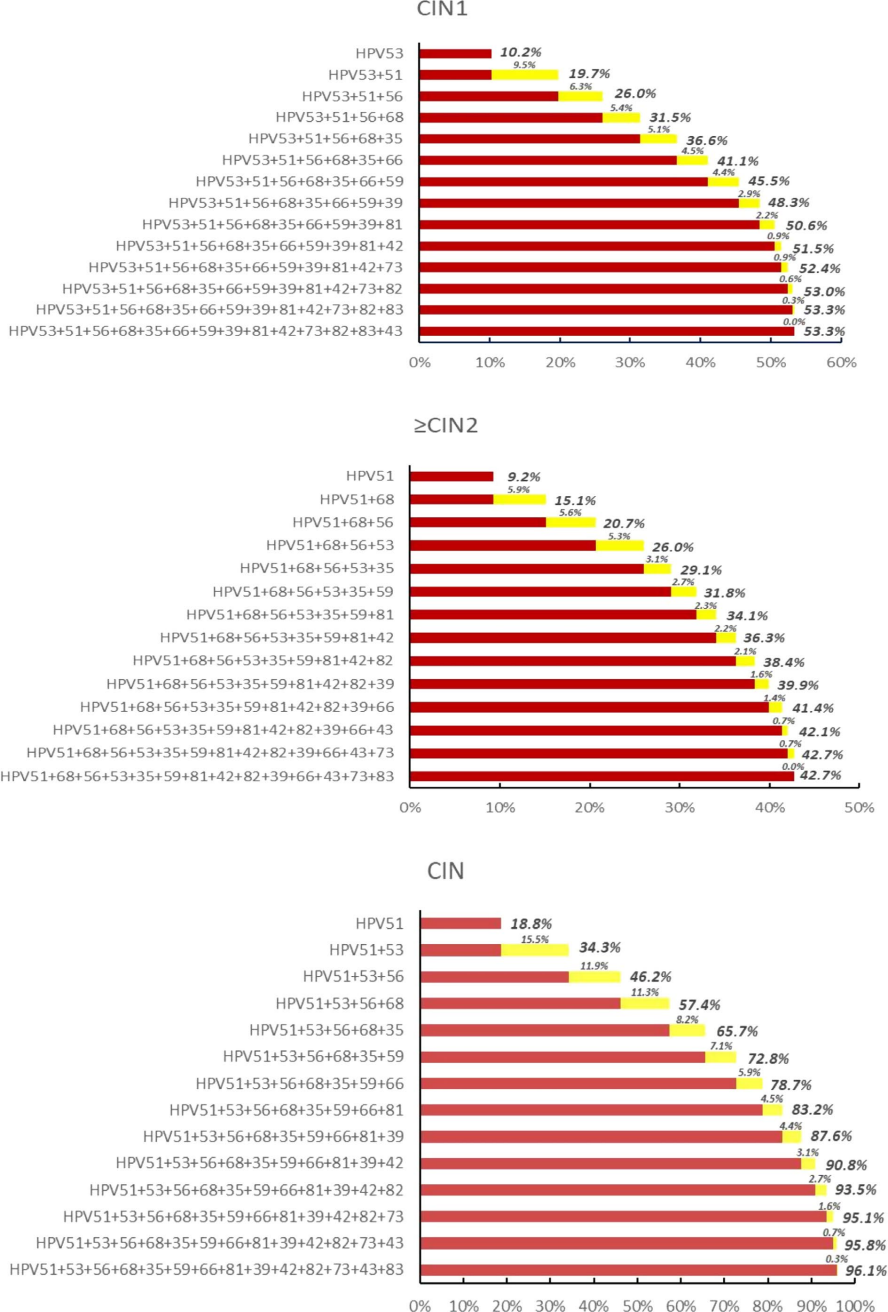
Cumulative attribution rate of 14 non‐9‐valent vaccine covered HPV subtypes in cervical lesions

In our cohort, the current non‐9‐valent vaccine covered HPV subtypes could be attributed to 53.3% of CIN1 and 42.7% of ≥CIN2. The CIN1 grade is mainly attributed to the HPV53 (10.2%), followed by HPV51 (9.5%) and 56 (6.2%). The ≥CIN2 grade is mainly attributed to HPV51 (9.2%), followed by HPV68 (5.9%) and 56 (5.6%). Among all CINs, the attribution rates of HPV51, 53, and 56 were 18.8%, 34.3%, and 46.2%, respectively. Meanwhile, HPV51, 53, 56, and 68 accounted for the majority of cervical lesions in each group, and the cumulative attribution rates were 31.5% in CIN1 and 26.0% in ≥CIN2 (Figure 1).

### 3.4 Distribution of multiple infection rates of non‐9‐valent vaccine covered HPV subtypes

In all patients infected with non‐9‐valent vaccine covered HPV subtypes, one, two, three, four subtypes, high‐risk non‐9‐valent vaccine covered, and low‐risk non‐9‐valent vaccine covered subtypes were detected with infection rates of 56.5%, 15.2%, 3.8%, 1.01%, 59.2%, and 43.9%, respectively. The rate of monotype infection was the highest in the normal group (59.0%) and lowest in ≥CIN2 group (37.9%). The infection rate of two subtypes of HPV increased with the aggravation of cervical lesions, and the infection rate of patients with ≥CIN2 was the highest (22.9%), while triple and quadruple infections were most common in the CIN1 group with infection rates of 5.0% and 1.2%, respectively. Furthermore, the infection rate of HPV subtypes covered by high‐risk non‐9‐valent vaccine was higher than that covered by low‐risk non‐9‐valent vaccine among all three groups of patients. Conversely, the infection rate of HPV subtypes covered by low‐risk non‐9‐valent vaccine decreased gradually. Consecutively, two subtypes of HPV infections were found to be risk factors for ≥CIN2 compared to the normal and CIN1 groups (normal, *p* = 0.001; CIN1, *p* = 0.034). Compared to the normal group, the HPV infection covered by high‐risk non‐9‐valent vaccine was a risk factor for ≥CIN2 (*p* = 0.027) (Table 3).

**TABLE 3:** Distribution of multiple infection rates of non‐9‐valent vaccine covered HPV subtypes

HPV type	Normal (*n* = 2179)	CIN1 (*n* = 338)	≥CIN2 (*n* = 153)	Total
	*n* (%)	*n* (%)	*n* (%)	*n* (%)
Single type	1286 (59.0)	165 (48.8)	58 (37.9)^a^	1509 (56.5)
Two types	320 (14.7)	52 (15.4)	35 (22.9)^b^	407 (15.2)
Three types	77 (3.5)	17 (5.0)	6 (3.9)^c^	100 (3.8)
Four types	22 (1.0)	4 (1.2)	1 (0.7)^d^	27 (1.0)
N9‐HR‐HPV	1274 (58.5)	210 (62.1)	97 (63.4)^e^	1581 (59.2)
N9‐LR‐HPV	980 (45.0)	124 (36.7)	52 (34.0)^f^	1156 (43.3)

Abbreviations: N9‐HR‐HPV, high‐risk HPV covered by non‐9‐valent vaccines, including HPV35/39/51/53/56/59/66/68/73/82; N9‐LR‐HPV, low‐risk HPV covered by non‐9‐valent vaccines, including HPV42/43/81/83.

^a^ The proportion of single type in ≥CIN2 group was not significantly different from that in CIN1 group (*p* > 0.05, χ^2^ = 2.704), but was significantly different from that in the normal group (*p* = 0.003, χ^2^ = 9.101).

^b^ The proportion of the two types of HPV infection in ≥CIN2 groups was significantly different from that in the other two groups (CIN1, *p* = 0.034, χ^2^ = 4.515; Normal, *p* = 0.001, χ^2^ = 10.821).

^c^ No significant difference was detected in the proportions of three types of HPV infection between ≥CIN2 group and other groups (CIN1, *p* > 0.05, χ^2^ = 0.202; Normal, *p* > 0.05, χ^2^ = 0.076).

^d^ No significant difference was detected in the proportions of four types of HPV infection between ≥CIN2 group and other groups (CIN1, *p* > 0.05, χ^2^ = 0.254; Normal, *p* > 0.05, χ^2^ = 0.001).

^e^ The proportion of HPV infection covered by N9‐HR‐HPV in the ≥CIN2 group was significantly different from that in the normal group (*p* < 0.027, χ^2^ = 4.897), but not significantly different from that in the CIN1 group (*p* > 0.05, χ^2^ = 0.373).

^f^ No significant difference was observed in the proportion of N9‐LR‐HPV between ≥CIN2 and other groups (CIN1, *p* > 0.05, χ^2^ = 0.065; Normal, *p* > 0.05, χ^2^ = 1.896).

### 3.5 Correlation between HPV subtype infection, clinical factors, and cervical lesions

Then, we analyzed the clinical risk factors of non‐9‐valent vaccines covered HPV‐infected CIN patients through single‐factor and multi‐factor. Multivariate analysis showed that HPV35, HPV53, HPV81, HPV83, menopause, and cervical transformation area were independent risk factors for CIN compared to the normal group (OR > 1, *p* < 0.05). Cervical transformation zone was an also critical risk factor (OR = 4.1). In patients with TCT ≥ atypical squamous cells of unknown significance (ASC‐US), HPV35, HPV42, HPV83, age, menopause, and cervical transformation zone were independent risk factors for CIN (Table 4). Meanwhile, compared to CIN1 group, HPV53, HPV81, age, menopause, cervical transformation area, and involved glands were independent risk factors for ≥CIN2 group. In patients with TCT ≥ ASC‐US, HPV51, HPV53, HPV68 infection, age, and cervical transition area were independent risk factors for ≥CIN2 (*p* < 0.05) (Table S2).

**TABLE 4:** Correlation between HPV subtype infection, clinical factors, and cervical lesions

Variables	Group 1 (*n* = 491)			Group 2 (*n* = 356)		
	OR	95% CI	*p* value^a^	OR	95% CI	*p* value^a^
HPV35	2.08	1.35–3.20	0.001	2.76	1.48–5.15	0.001
HPV39	1.19	0.77–1.84	0.433	1.19	0.66–2.14	0.573
HPV42	1.70	0.99–2.94	0.057	2.43	1.09–5.41	0.03
HPV43	1.66	0.94–2.93	0.083	1.91	0.93–3.93	0.08
HPV51	0.98	0.71–1.36	0.924	1.06	0.68–1.65	0.797
HPV53	1.55	1.12–2.15	0.008	1.38	0.886–2.155	0.154
HPV56	1.07	0.76–1.49	0.706	1.06	0.67–1.68	0.813
HPV59	1.00	0.64–1.55	0.982	1.72	0.95–3.13	0.075
HPV66	1.28	0.82–2.01	0.274	0.60	0.32–1.14	0.121
HPV68	0.79	0.54–1.16	0.226	1.48	0.80–2.75	0.214
HPV73	1.07	0.46–2.52	0.875	1.13	0.34–3.75	0.841
HPV81	2.08	1.30–3.34	0.003	0.60	0.34–1.08	0.087
HPV82	2.06	0.94–4.53	0.072	0.37	0.12–1.16	0.087
HPV83	2.17	1.70–10.29	0.002	2.87	1.16–12.92	0.028
Age	1.01	0.99–1.03	0.12	1.98	1.76–3.00	0.045
Pregnancy	1.04	0.95–1.13	0.424	1.06	0.94–1.19	0.323
Parity	1.06	0.90–1.24	0.482	1.17	0.95–1.45	0.148
Condom	1.01	0.76–1.33	0.971	1.07	0.75–1.54	0.716
Menopause	1.52	1.36–3.77	0.002	1.45	1.07–2.77	0.003
Cervical transformation area	4.08	3.24–5.14	<0.001	1.69	1.30–2.19	<0.001

Note

Group 1, the CIN group with HPV subtype infection was covered by non‐9‐valent vaccine; Group 2, CIN group with HPV subtype infection covered by non‐9‐valent vaccine in patients with TCT ≥ ASC‐US.

^a^ The risk of cervical disease in each group was calculated with reference to the corresponding normal cervical group.

### 3.6 Analysis of infection rate of non‐9‐valent vaccine covered HPV subtypes in different age groups

**FIGURE 2:**
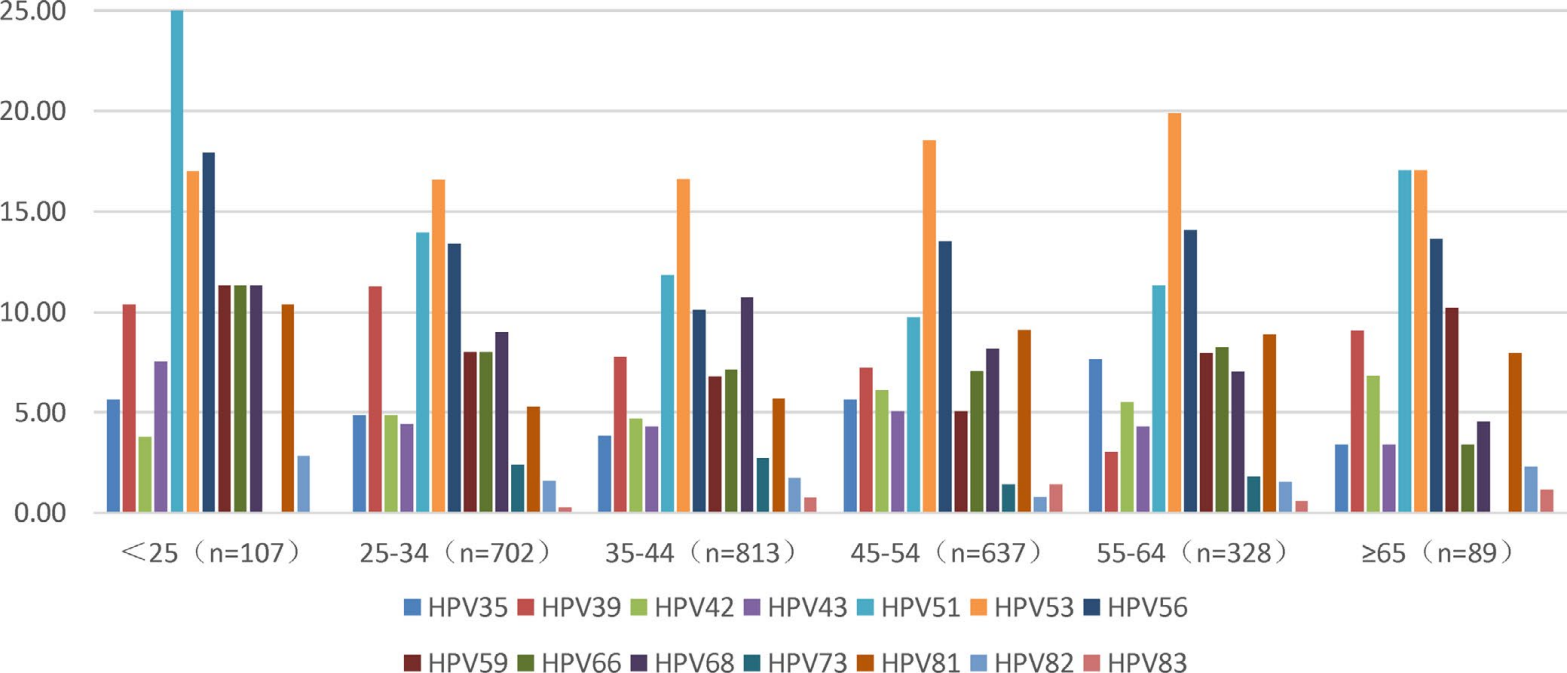
Analysis of infection rate of HPV subtypes in different age groups

**FIGURE 3:**
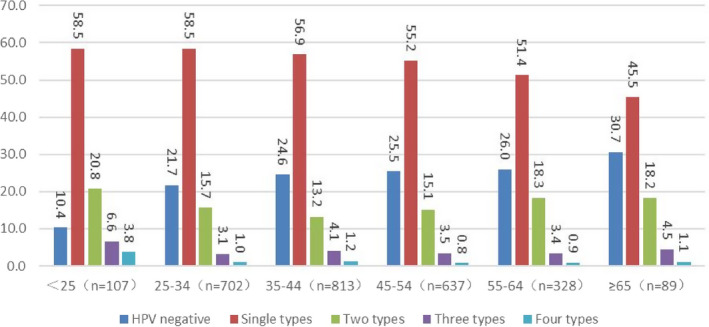
Analysis of the infection rates of single and multiple non‐9‐valent HPV subtypes in different age groups

Finally, we analyzed age‐stratified HPV distribution of the subjects. Among the individuals <25 years old, the infection rate of HPV51 was the highest (25.5%). Among the patients aged 25–34, 35–44, 45–54, and 55–64 years, the HPV subtype with the highest infection rate was HPV53. Among the patients ≥65 years old, the infection rates of HPV51 and 53 were the highest at 17.1% (Figure 2). In all age groups, compared to the multiple non‐9‐valent vaccine covered HPV subtypes infections, the infection rate of single HPV was the highest, and with the increase in age, the infection rate decreased gradually. For patients with double (20.8%) and triple (6.6%) HPV infections, the highest infection rate was observed in <25 years old, while the lower infection rate was found in 35–44 years old (13.2%) and 25–34 years old (3.1%). In addition, HPV‐negative was common in the age group ≥65 years old, accounting for 30.7% of cases (Figure 3).

## 4 DISCUSSION

Deaths from cervical cancer could be prevented through regular screening and early treatment, as well as vaccination against HPV.^15^ Although expanding HPV vaccination can prevent more cervical cancer,^16^ the current coverage in low‐and middle‐income countries is yet low.^17^ Moreover, the existing multivalent vaccines contain only a fraction of HR‐HPV subtypes. Therefore, it is crucial to understand the distribution and risk factors of non‐9‐valent vaccine covered HPV genotypes in the post‐vaccine era to prevent and treat cervical lesions.

Herein, we showed that pregnancy ≥5 times, delivery ≥3 times, and non‐use of condoms were risk factors for cervical lesions that were consistent with those from previous studies.^18^ Although previous studies showed that oral contraceptives and intrauterine device (IUD) could increase the risk of cervical cancer,^18^, ^19^ our present study found that other contraceptives other than condoms had no statistical significance among all groups, which might be related to the small sample size. While previous studies have shown that the most precancerous lesions and cancer occur in the cervical transformation area,^20^ we found that cervical transformation zone type III is a risk factor for CIN1 or ≥CIN2, which is consistent with the study of Jin et al.^21^

Wang et al.^22^ demonstrated that the most common non‐9‐valent vaccine covered HPV subtypes in cervical lesions were HPV53, 68, and 39. In our current cohort, we found that HPV53, 56, 51, and 68 had higher infection rates among 14 HPV subtypes covered by non‐9‐valent vaccine, while previous studies showed that HPV51, 53, 56, 59, 66, and 68 were associated with CIN2/3 and invasive cervical squamous cell carcinoma (SCC).^23^ A meta‐analysis showed that the most common non‐9‐valent vaccine covered HPV subtype in the histological high‐grade squamous intraepithelial lesion (HSIL) group was 51 and 56 in Asia.^24^ Another study showed that the most common non‐9‐valent vaccine covered HPV subtype was HPV39 in CIN1, HPV51 in CIN2, and CIN3,^25^ and the infection rate of HPV39 and 51 increased significantly in recent years.^26^ In our study, the infection rates of HPV53, 56, and 51 were higher in CIN1 group, while in the patients with ≥CIN2, the infection rates of HPV56, 51, and 53 were higher, which might be related to the differences in the distribution of HPV in various regions and populations. Multivariate analysis showed that HPV35, HPV53, HPV81, and HPV83 were independent risk factors for CIN compared to the normal group. Although the 9‐valent HPV vaccine is expected to prevent 90% of cervical cancer, except for the nine types covered by the vaccine, the 9‐valent HPV vaccine does not prevent other HPV‐related infections and diseases.^27^ Moreover, some studies have shown that the common HPV subtypes in elderly women include HPV61, 71, 72, 81, 83, 84, and 89.^28^ HPV81 and 83 are risk factors for cervical intraepithelial neoplasia in elderly women.^29^ Therefore, combined with the results of previous studies, it is suggested that we should pay more attention to the infection of HPV35, 53, 51, 56, 81, and 83 in the post‐vaccine era, and these HPV subtypes could be covered in future vaccine preparation. Nonetheless, the risk distribution of HPV81 and 83 also needs to be analyzed in future studies.

Previous studies have shown that the cumulative attribution rate of high‐risk HPV subtypes covered by 9‐valent vaccine was 38.4% in the low‐grade squamous intraepithelial lesion (LSIL) group and 68.4% in the HSIL group.^30^ Here, we found that the cumulative attribution rate of non‐9‐valent vaccine covered HPV subtypes was 53.3% in CIN1, which was higher than that in ≥CIN2 (42.7%). This phenomenon further confirmed that cytological screening is greatly affected in the vaccinated population, because the surplus of low‐grade lesions is elevated.^31^ Also, while the 9‐valent vaccine was widely used, the non‐9‐valent vaccine covered HPV subtypes exhibited high morbidity.

The attribution rate of HPV51 in CIN1 (9.5%) and ≥CIN2 (9.2%) in current study was higher than that of the findings of Chan et al.,^14^ which might be related to varied study areas and populations. We also found that 31.47% of CIN1 and 26.0% of ≥CIN2 were attributed to HPV51, 53, 56, and 68. Therefore, it is suggested that these HPV subtypes could be included in future vaccine research.

Diverse from the results of the previous study,^22^ we found that the haplotype infection rate was the highest in the normal group and the lowest in the ≥CIN2 group. The comparison between groups showed that double HPV infection was a risk factor for ≥CIN2, indicating that double HPV infection has a significant risk and promotes the occurrence and development of cervical lesions and cancer.^32^ Consecutively, we found that the more severe the cervical lesions, the higher the rate of N9‐HR‐HPV. Moreover, the N9‐HR‐HPV is also a risk factor ≥CIN2. Therefore, for women who have been vaccinated with 9‐valent vaccine, cervical cancer screening is still essential.

The current study found that the higher infection rates of HPV subtypes not covered with 9‐valent vaccine in different age groups were HPV51, 56, and 53. A previous study found that the most common non‐9‐valent vaccine coverage HPV genotypes in women <50‐years‐old were HPV59, 39, and 56, while in elderly women >50 years old, the most common HPV subtypes were 56 and 68,^33^ which might be related to different regions and target populations of the study. For patients with multiple HPV infection, the age group with the highest infection rate is all <25‐year‐old, which is consistent with previous studies.^34^

Since the current study showed that some non‐9‐valent vaccine covered HPV subtypes was vigilant for the risk of cervical lesions, we still need to focus on the cervical precancerous lesions and cervical cancer caused by non‐9‐valent vaccines HPV subtypes (including HPV‐negative). In the case of women who have been vaccinated, we should also strengthen the universal education and pay attention to the non‐9‐valent vaccine covered HPV subtypes, such as HPV35, 51, 53, 56, 81, and 83. In addition, with the change in social population structure and increased aging, for elderly women, if there is low‐risk HPV (such as 81 and 83) infection, it still needs to be considered in combination with specific clinical conditions.

Nevertheless, there are some deficiencies in the present study. First, the retrospective study is easily affected by the selection offset. Second, we lack data on the other risk factors for patients with non‐9‐vaccine covered HPV subtypes, including smoking volume, economic status, educational level, number of sexual partners, family history, etc.

In conclusion, our study analyzed the infection rate of non‐9‐valent vaccines covered HPV subtypes in cervical lesions. The clinical characteristics and risk factors of CIN patients with non‐9‐valent vaccines covered HPV subtypes were also explored. The results suggest that sufficient follow‐up of future studies with a larger sample size can further assess the carcinogenic risk of non‐9‐valent vaccines HPV covered subtypes, which might be helpful to optimize the screening and prevention strategies of cervical cancer in the post‐vaccine era.

## ETHICS STATEMENT

The study is approved by the Ethics Committee of the Shanghai General Hospital (reference 2021SQ263). As all analyses were performed on pseudonymized and already collected data, written consent from participants was not required.

## CONFLICT OF INTEREST

The authors have no potential conflict (financial, professional, or personal) to disclose in terms of this manuscript.

## AUTHOR CONTRIBUTION

S Wu and J Zhang conceived and designed the whole project and drafted the manuscript. M Ma, J Zhu, and Y Yang analyzed the data and carried out data interpretations. X Wang and Y Jin helped data discussion. M Ma, J Zhu, and Y Yang wrote the manuscript. J Zhang revised the manuscript. All authors have read and approved the final copy.

## Supporting information

Table S1

Table S2

## Data Availability

Raw data were obtained from Department of Gynecology and Obstetrics, Shanghai General Hospital, Shanghai Jiao Tong University School of Medicine. The data that support the findings of this study are available from the corresponding author, upon reasonable request.
