# Supplementary material for: The distribution and pathogenic risk of non‐9‐valent vaccine covered HPV subtypes in cervical lesions
Source: Cancer Med. 2022 Jan 3;11(6):1542–52. doi: 10.1002/cam4.4532 (PMC8921916; doi:10.1002/cam4.4532)
Supplement: Supplementary file 1 — Table S1 [file CAM4-11-1542-s001.docx]

| **Supplementary Table S1.**  Analysis of infection in single- and multi-type non-9-valent vaccine covered HPV subtypes | | | | | | | | | | | | |
| --- | --- | --- | --- | --- | --- | --- | --- | --- | --- | --- | --- | --- |
| HPV types | Normal （n=5751） | | | CIN1(n=856) | | | ≥CIN2(n=793) | | | Total | | |
|  | positive n(%^a^） | Single type n(%^a^） | Multiple infections n(%^a^） | positive n(%^a^） | Single type n(%^a^） | Multiple infections n(%^a^） | positive n(%^a^） | Single type n(%^a^） | Multiple infections n(%^a^） | positive n(%^a^） | Single type n(%^a^） | Multiple infections n(%^a^） |
| HPV35 | 153(2.7) | 84(1.5) | 69(1.2) | 41(4.8) | 17(2.0) | 24(2.8) | 17(2.1) | 4(0.5) | 13(1.6) | 211(2.9) | 105(1.4) | 106(1.4) |
| HPV39 | 284(4.9) | 127(2.2) | 157(2.7) | 29(3.4) | 9(1.1) | 20(2.3) | 15(1.9) | 3(0.4) | 12(1.5) | 328(4.4) | 139(1.9) | 189(2.6) |
| HPV42 | 288(5.0) | 83(1.4) | 205（3.6） | 16(1.9) | 5(0.6) | 11(1.3) | 12(1.5) | 3(0.4) | 9(1.1) | 316(4.3) | 91(1.2) | 225(3.0) |
| HPV43 | 155(2.7) | 71(1.2) | 84（1.5） | 14(1.6) | 2(0.2) | 12(1.4) | 6(0.8) | 1(0.1) | 5(0.6) | 175(2.4) | 74(1.0) | 101(1.4) |
| HPV51 | 445(7.7) | 186(3.2) | 259（4.5） | 68(7.9) | 32(3.7) | 36(4.2) | 38(4.8) | 14(1.8) | 24(3.0) | 551(7.5) | 232(3.1) | 319(4.3) |
| HPV53 | 633(11.0) | 300(5.2) | 333（5.8） | 76(8.9) | 32(3.7) | 44(5.1) | 36(4.5) | 7(0.9) | 29(3.7) | 745(10.1) | 339(4.6) | 406(5.5) |
| HPV56 | 354(6.2) | 201(3.5) | 153（2.7） | 69(8.1) | 20(2.3) | 49(5.7) | 25(3.2) | 10(1.3) | 15(1.9) | 448(6.1) | 231(3.1) | 217(2.9) |
| HPV59 | 256(4.5) | 96(1.7) | 160（2.8） | 42(4.9) | 14(1.6) | 28(3.3) | 14(1.8) | 4(0.5) | 10(1.3) | 312(4.2) | 114(1.5) | 198(2.7) |
| HPV66 | 277(4.8) | 111(1.9) | 166（2.9） | 36(4.2) | 17(2.0) | 19(2.2) | 14(1.8) | 3(0.4) | 11(1.4) | 327(4.4) | 131(1.8) | 196(2.7) |
| HPV68 | 265(4.6) | 157(2.7) | 108（1.9） | 34(4.0) | 17(2.0) | 17(2.0) | 22(2.8) | 7(0.9) | 15(1.9) | 321(4.3) | 181(2.4) | 140(1.9) |
| HPV73 | 76(1.3) | 25(0.4) | 51（0.9） | 10(1.2) | 5(0.6) | 5(0.6) | 4(0.5) | 1(0.1) | 3(0.4) | 90(1.2) | 31(0.4) | 59(0.8) |
| HPV81 | 226(3.9) | 92(1.6) | 134（2.3） | 28(3.3) | 7(0.8) | 21(2.5) | 22(2.8) | 4(0.5) | 18(2.3) | 276(3.7) | 103(1.4) | 173(2.3) |
| HPV82 | 51(0.9) | 13(0.2) | 38（0.7） | 10(1.2) | 3(0.4) | 7(0.8) | 7(0.9) | 3(0.4) | 4(0.5) | 68(0.9) | 19(0.3) | 49(0.7) |
| HPV83 | 23(0.4) | 3(0.1) | 20（0.4） | 6(0.7) | 1(0.1) | 5(0.6) | 4(0.5) | 0(0.0) | 4(0.5) | 33(0.5) | 4(0.1) | 29(0.4) |

*Notes*: ^a^HPV subtype infection rate, it was calculated by dividing the number of women infected with HPV by the total number of participants in the study.
