# Supplementary material for: The distribution and pathogenic risk of non‐9‐valent vaccine covered HPV subtypes in cervical lesions
Source: Cancer Med. 2022 Jan 3;11(6):1542–52. doi: 10.1002/cam4.4532 (PMC8921916; doi:10.1002/cam4.4532)
Supplement: Supplementary file 2 — Table S2 [file CAM4-11-1542-s002.docx]

| **Supplementary Table S2.** Correlation between HPV subtype infection, clinical factors and cervical lesions | | | | | | | |
| --- | --- | --- | --- | --- | --- | --- | --- |
| Variables | Group1 (n=153) | | |  | Group 2 (n=128) | | |
|  | OR | 95%CI | *P value*^a^ |  | OR | 95%CI | *P value*^a^ |
| HPV35 | 1.66 | 0.66-4.14 | 0.28 |  | 0.89 | 0.30-2.69 | 0.842 |
| HPV39 | 1.38 | 0.54-3.50 | 0.501 |  | 1.55 | 0.47-5.14 | 0.472 |
| HPV42 | 2.23 | 0.65-7.62 | 0.203 |  | 1.59 | 0.25-9.90 | 0.622 |
| HPV43 | 1.66 | 0.48-5.75 | 0.425 |  | 1.03 | 0.23-4.65 | 0.973 |
| HPV51 | 1.42 | 0.75-2.68 | 0.282 |  | 2.20 | 1.01-4.79 | 0.048 |
| HPV53 | 4.68 | 1.88-11.66 | 0.001 |  | 6.03 | 2.01-18.09 | 0.001 |
| HPV56 | 2.18 | 0.96-4.93 | 0.062 |  | 2.26 | 0.79-6.41 | 0.127 |
| HPV59 | 2.63 | 0.96-7.20 | 0.059 |  | 2.34 | 0.72-7.57 | 0.155 |
| HPV66 | <0.001 | <0.001 | 0.998 |  | <0.001 | <0.001 | 0.998 |
| HPV68 | 1.43 | 0.66-3.09 | 0.369 |  | 3.05 | 1.06-8.79 | 0.039 |
| HPV73 | <0.001 | <0.001 | 0.999 |  | <0.001 | <0.001 | 0.999 |
| HPV81 | 5.12 | 1.38-19.05 | 0.015 |  | 3.86 | 0.92-16.28 | 0.066 |
| HPV82 | 1.00 | 0.23-4.45 | 0.998 |  | 0.63 | 0.11-3.55 | 0.602 |
| HPV83 | 5.45 | 1.27-23.35 | 0.022 |  | 4.69 | 0.76-28.75 | 0.095 |
| Age | 1.94 | 1.91-3.97 | <0.001 |  | 1.91 | 1.87-3.95 | <0.001 |
| Pregnancy | 1.14 | 0.95-1.36 | 0.161 |  | 1.10 | 0.89-1.36 | 0.363 |
| Parity | 1.26 | 0.94-1.69 | 0.125 |  | 1.41 | 0.98-2.02 | 0.065 |
| Condom | 1.34 | 0.74-2.44 | 0.333 |  | 1.82 | 0.90-3.69 | 0.097 |
| Menopause | 1.35 | 1.15-2.80 | 0.013 |  | 1.11 | 1.04-3.33 | <0.001 |
| Cervical transformation area | 1.93 | 1.13-3.31 | 0.016 |  | 7.52 | 3.81-14.83 | <0.001 |
| Involved glands | 2.20 | 1.11-3.71 | 0.021 |  | 1.22 | 0.73-2.04 | 0.445 |

Notes: Gourp 1, non-9-valent vaccine covered HPV subtypes infection ≥CIN2 group; Group 2, among patients with TCT ≥ ASC-US, non-9-valent vaccine covered HPV subtype infection ≥ CIN2 group.

^a^ The risk of cervical disease in each group was calculated with reference to the corresponding CIN1.
